# Gum Arabic/Gelatin and Water-Soluble Soy Polysaccharides/Gelatin Blend Films as Carriers of Astaxanthin—A Comparative Study of the Kinetics of Release and Antioxidant Properties

**DOI:** 10.3390/polym13071062

**Published:** 2021-03-28

**Authors:** Katarzyna Łupina, Dariusz Kowalczyk, Waldemar Kazimierczak

**Affiliations:** 1 Department of Biochemistry and Food Chemistry, Faculty of Food Sciences and Biotechnology, University of Life Sciences in Lublin, Skromna 8, 20-704 Lublin, Poland; katarzyna.lupina@gmail.com; 2 Laboratory of Biocontrol, Application and Production of EPN, Faculty of Natural Sciences and Health, Center for Interdisciplinary Research, John Paul II Catholic University of Lublin, Konstantynów 1J, 20-708 Lublin, Poland; waldemar.kazimierczak@kul.pl

**Keywords:** edible films, gum arabic, water-soluble soy polysaccharides, gelatin, FTIR, solubility, swelling, release rate, mathematical models, 2,2′-Azino-bis(3-ethylbenzothiazoline-6-sulfonic acid) diammonium salt (ABTS)

## Abstract

Polymer blending and incorporation of active substances offer a possibility of generation of novel packaging materials with interesting features. Astaxanthin is one of the most powerful antioxidants. Hence, in this study, water-soluble AstaSana astaxanthin (AST) was incorporated into 75/25 gum arabic/gelatin (GAR75/GEL25) and water-soluble soy polysaccharides/gelatin (WSSP75/GEL25) blend films in different concentrations (0, 0.25%, 0.5%, 1%). Microscope images showed good compatibility between the polysaccharides and GEL. Basing on time required for 50% release, the WSSP-based film exhibited an approximately four-fold slower release rate (t_50%_ = 65.16–142.80 min) than the GAR-based film (t_50%_ = 14.64–34.02 min). This result was mainly ascribed to the slower dissolution of the WSSP-based carrier. The faster release rate of the GAR-based films resulted in stronger antioxidant activity (quarter-scavenging time (t_25%ABTS_) = 0.22–7.51 min) in comparison to the WSSP-based films (t_25%ABTS_ = 0.91–12.94 min). The increase in the AST concentration was accompanied by gradually reduced solubility and the release rate. It is possible that the increasing number of starch granules (from the AST formulation) acted as a dissolution blocking agent. In general, the WSSP75/GEL25 film displayed the most linear (the Zero-order similar) release profile. So, this carrier has potential for release of AST at a quasi-constant speed.

## 1. Introduction

The growing demand for biodegradable packaging formats/types has increased the interest in eco-friendly films prepared from polysaccharides, proteins, and their blends. This type of materials can be applied as thin edible coatings, casing, wraps, layers separating various components in complex food products, dosage forms for packaging pre-weighed quantities of food, etc. Moreover, biopolymeric films can be used as carriers of a wide range of food preservatives. Antioxidant packaging is designed to delay food deterioration by slow release of antioxidants from the packaging material into the food surface, where the oxidation reactions mainly occur [1]. The designed migration of antioxidants from the active packaging system is a key determinant of its effectiveness in contact with water-rich foods. There are many mechanisms by which the release of active substances can be controlled in a system: dissolution, diffusion, osmosis, swelling, and erosion. They are dependent on the carrier type and may act simultaneously or at different stages of a migration process [2,3]. Biopolymers differ in their water affinities and, consequently, release profiles [4,5]. For instance, the water-swollen gelatin (GEL) films can be fabricated for controlled-release purposes, while the fast-dissolving carrier matrices based on linear polysaccharides offer quick release (“burst effect”) [6,7]. The release rate should closely match the requirement of the kinetics of food spoilage. Too slow a rate would result in an insufficient amount of the active compounds to retard food deterioration, whereas too fast a rate would result in an excessive amount and loss of the active compounds due to degradation [8].

Branched polymers, due to a large variety of available shapes, topologies, and compositions, have numerous and various applications. Compared to linear polymers, branched polymers display lower density due to the reduced packing efficiency of their chains. This imparts a number of favorable properties, including low intrinsic viscosities, high solubilities, globular conformation, and high surface functionality (the high number of functionalizable groups on the periphery of the constructs), which allow drug attachment [9]. Currently, branched, grafted, dendritic, and star polymers are being intensively explored as future carriers owing to advances in polymer chemistry [10].

Hemicelluloses (a mixture of highly branched low-molecular-weight homo- and heteropolymers) are the second most abundant polysaccharides in nature, representing about 20–35% of lignocellulosic biomass [11]. Hemicelluloses can be novel promising vehicles for delayed release of food additives. In support, a recent study conducted by Łupina, Kowalczyk, and Drozłowska [12] showed that hemicelluloses, such as gum arabic (GAR) and water-soluble soy polysaccharides (WSSP), ensured a controlled rate of lipophilic vitamin C release. The GAR- and soy polysaccharide-based systems have also been used for targeted and sustained release of various pharmacological substances. Unfortunately, branching makes the polymers less dense, which results in low mechanical strength. However, reinforcement of hemicelluloses with stronger polymers, such as GEL and chitosan [13,14], can overcome this problem.

Carotenoids have been described not only as coloring agents but also as antioxidant compounds. Astaxanthin (ASX) is a pigment with higher antioxidant activity than other known natural antioxidants. ASX is contained in green algae *Haematococcus pluvialis* and red yeast *Phaffia rodozyma* [15]. The oldest approach to obtain carotenoids is extraction from biological material [16]. The main disadvantage of this production method is the high cost, geographic determinants, and seasonality of the raw material. Moreover, natural carotenoids exhibit low resistance to external factors, a variable dye composition, and it is not easy to standardize their color. Chemical synthesis has been used in production of carotenoids since 1950. Of the nearly 700 naturally occurring carotenoids, only a few are synthesized on an industrial scale. Among them is ASX [17].

The available synthetic forms of ASX esters differ in their molecular profile from ASX obtained from extracts of living organisms [4]. First of all, synthetic ASX is an exclusively “free” compound (i.e., it is non-esterified and has no fatty acids attached to the ends of the molecule). Moreover, it contains no supporting carotenoids. Therefore, synthetic ASX has lower antioxidant potential than the natural one [18]. Nevertheless, the synthetic compound is much cheaper (US$2500/kg) than the natural one (>US$7000/kg) [19]. Consequently, over 95% of the ASX available on the market is produced synthetically, whereas natural ASX from *Haematococcus* represents <1% of the commercial product [20].

The 11 conjugated double bonds in the structure of ASX make it unstable, which seriously restricts its applications in functional foods and pharmaceutical and cosmetic products [9]. Addition of co-antioxidants to the ASX formulation reduces its decomposition, thus, this practice is commonly applied by large chemical companies.

The scientific literature is increasingly reporting the antioxidant and pro-health effects of synthetic ASX. Santocomo et al. [21] showed that synthetic ASX was capable of protecting the DNA of neuroblastoma cells exposed to reactive nitrogen species. Also, in human dermal fibroblasts exposed to moderate doses of UVA, synthetic ASX exhibited a pronounced photoprotective effect. In comparison with irradiated control cells, the formation of thiobarbituric acid reactive substances decreased to approximately 70% [22]. Gross and Lockwood [23] found that the water-dispersible disodium disuccinate derivative of synthetic ASX (CardaxTM) exhibited a cardioprotective effect in Sprague Dawley rats.

The poor water solubility of natural ASX greatly reduces its bioavailability, which also has a negative effect on its practical applications. Therefore, the aim of this study was to characterize the release and antioxidant properties of binary 75/25 GAR/GEL (GAR75/GEL25) and WSSP/GEL (WSSP75/GEL25) blend films incorporated with increasing concentrations (0, 0.25, 0.5, 1%) of synthetic water-soluble AstaSana ASX (AST). The microstructure, pH, and water affinities (dissolution and swelling) of the films were determined to provide insight into the AST release mechanisms.

To the best of our knowledge, this is the first study on the release of water-soluble ASX from active packaging films.

## 2. Materials and Methods

### 2.1. Materials

Pork GEL (with a bloom strength of 240; McCormick-Kamis S.A., Stefanowo, Poland), GAR (Agri-Spray Acacia R, Agrigum International, Old Amersham, UK), WSSP (Gushen Biological Technology Group Co., LTD, Dezhou, China), glycerol (Sigma Chemical Co., St. Louis, MO, USA), and AST (gifted from DSM, Heerlen, Netherlands; composition: ASX, modified starch, glucose syrup, dl-α-tocopherol, and sodium ascorbate) were used for film preparation. 2,2′-Azino-bis(3-ethylbenzothiazoline-6-sulfonic acid) diammonium salt (ABTS) was purchased from Sigma Chemical Co., St. Louis, MO (USA).

### 2.2. Film Preparation

The film-forming solutions (FFSs) were composed of a polysaccharide/GEL blend (5% *w/w*), glycerol (1% *w/w*), and increasing amounts of AST (0, 0.25, 0.5, 1% *w/w*). Powder polysaccharide/GEL 75/25 blends (3.75 g of polysaccharide blended with 1.25 g of GEL) were mixed with water (94 g) and glycerol (1 g), and then heated in a water bath at 90 °C for 1 h with constant stirring. The FFSs were cooled (to ~40 °C) and the AST was subsequently added (at the expense of reducing the amount of water). After degassing, the FFSs were placed on polycarbonate trays with an area of 4 cm^2^. A constant amount of total solids (0.0125 g/cm^2^) was placed on the tray to keep the film thickness. The FFSs were dried at 25 ± 2 °C and 50 ± 5% relative humidity (RH) for 24 h.

### 2.3. pH

A glass electrode (Elmetron ERH-11S, Zabrze, Poland) connected to a pH meter (Elmetron CPC 401, Zabrze, Poland) was used for measurement of pH of the FFSs at 40 °C. The analyses were performed in triplicate.

### 2.4. Film Thickness

The thickness of the film samples was determined using a micrometer (Mitotuyo 547-401, Tokyo, Japan).

### 2.5. Film Conditioning

The film samples were conditioned (50% RH, 25 °C, 48 h) in a test chamber (MLR-350, Sanyo Electric Biomedical Co. Ltd., Osaka, Japan).

### 2.6. Microscopy

The FFSs were examined using an inverted microscope (Olympus CKX53, Tokyo, Japan), a polarized light microscope LEICA 5500B (Leica Microsystems GmbH, Wetzlar, Germany), and a scanning electron microscope Carl Zeiss Ultra Plus (Oberkochen, Germany) using a cryogenic technique (cryo-SEM).

### 2.7. Attenuated Total Reflectance Fourier Transform Infrared (ATR-FTIR) Spectroscopy

FTIR tests were carried out using a Thermo Nicolet 8700 FTIR spectrometer (Thermo Scientific, Waltham, Massachusetts, USA) with a Smart Orbit accessory.

### 2.8. Water Affinities

The total soluble matter (TSM) was expressed as the percentage of film dry matter solubilized after immersion in water. The film specimens (4 cm^2^) were weighed (±0.0001 g) and shaken with distilled water (25 mL, 30 ± 1 °C, and 170 rpm) using a shaking incubator (ES-60, MIULAB, Hangzhou, China). Then, undissolved residues taken at different time points were removed from the water and dried (105 °C, 24 h). The initial dry matter content of the films, necessary for calculation of the TSM, was determined by drying at 105° for 24 h. Swelling (Sw) was evaluated by immersing the pre-weighed films (4 cm^2^) in distilled water (25 mL, 30 ± 1 °C, 1 min). The weight of swollen films was measured, after blotting the surface gently with filter paper until equilibrium was reached. Sw was calculated as the percentage of water absorbed by the sample. The tests were performed in triplicate.

### 2.9. Release Test

The film discs (4 cm^2^) were shaken with distilled water (25 mL, 30 ± 1 °C, 170 rpm, 4 h) in the shaking incubator. Two hundred and fifty microliters of the release media samples were taken at different time points and the absorbance was read at 464 nm using a microplate spectrophotometer (EPOCH 2 Microplate Spectrophotometer, BioTek, Winooski, USA). The analyses were performed in triplicate. DDSolver, i.e., add-in software for Microsoft Excel, was used for modeling the AST release kinetics. Ten mathematical models were chosen to fit the experimental data. The Akaike Information Criterion (AIC) was used for selection of the optimal mathematical models, which were used for determination of the quarter- and half-release times (t_25%_ and t_50%_). A lower AIC value indicates a better fit. The adjusted coefficient of determination (R^2^_adjusted_) values were estimated in order to present the accuracy of the t_25%_ and t_50%_ calculations [24].

### 2.10. Antiradical Activity

The film discs (4 cm^2^) were shaken with ABTS reagent (25 mL, 30 ± 1 °C, 170 rpm) in the shaking incubator. Two hundred and fifty microliters of the mixture samples were taken at different time points and the absorbance was read at 734 nm using a spectrophotometer (Lambda 40, Perkin–Elmer, Shelton, CT, USA). The absorbance was measured until the reaction reached a plateau (3 h). The ability of the films to quench ABTS free radicals was calculated using Equation (1).

Scavenging% = [1 – (Abs/Abs_ABTS_)] × 100 (1)

where Abs is the absorbance of the sample and Abs_ABTS_ is the absorbance of the ABTS solution (0.70 ± 0.05). The tests were performed in triplicate.

The Weibull with F_max_ (Wb F_max_) model was used for determination of quarter- and half-scavenging times (t_25%ABTS_ and t_50%ABTS_). The values of the adjusted coefficient of determination (R^2^_adjusted_) were estimated in order to present the accuracy of the t_25%ABTS_ and t_50% ABTS_ calculations.

### 2.11. Statistical Analysis

Differences among the data mean values were tested for statistical significance at the *p* < 0.05 level using analysis of variance (STATISTICA 13.1, StatSoft Inc., Tulsa, USA) and Fisher’s test. The data were evaluated using Pearson’s correlation coefficients to identify relationships between the TSM, the AST release, and the ABTS free radical scavenging ability.

## 3. Results and Discussion

### 3.1. Microstructure

**Figure 1:**
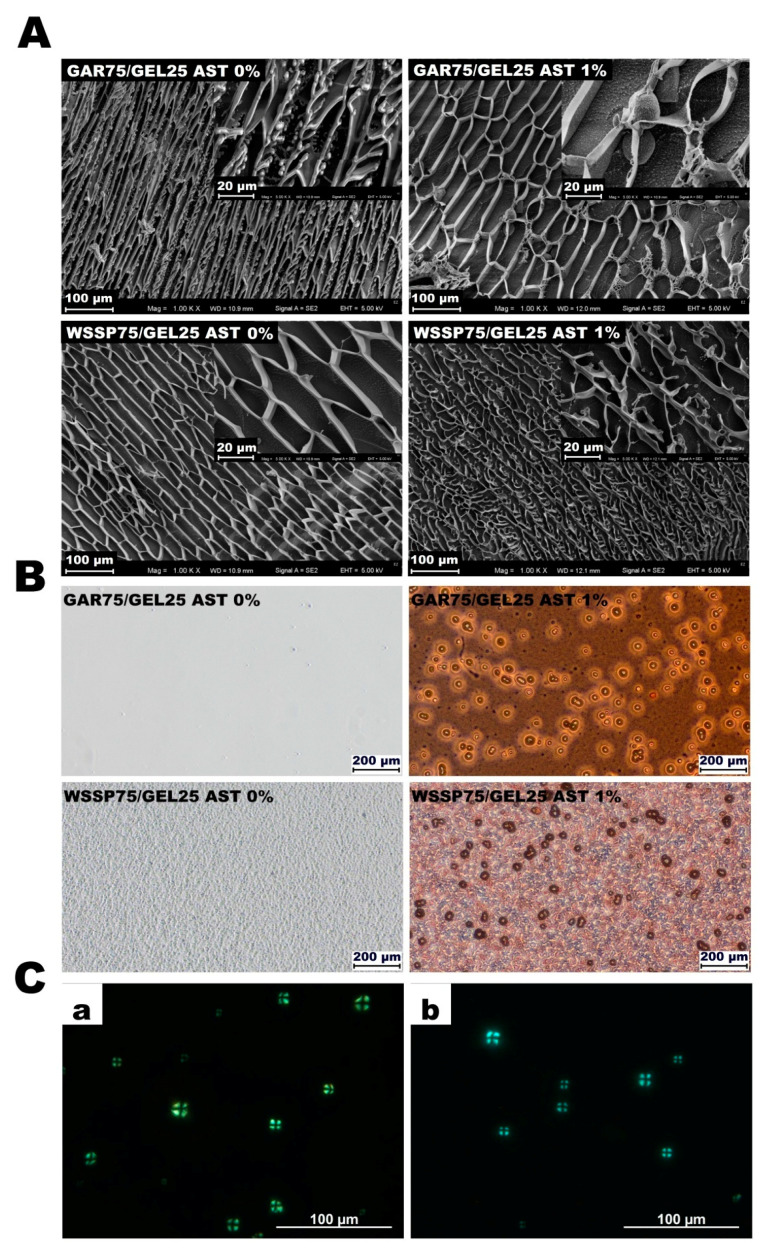
Images of AstaSana astaxanthin (AST)-free and 1%AST-supplemented film-forming solutions examined using the cryo-SEM technique (**A**) and the light microscope (**B**). Images of 0.5% AST-supplemented 75/25 gum arabic/gelatin (GAR75/GEL25) (**a**) and water-soluble soy polysaccharides/gelatin (WSSP75/GEL25) film-forming solutions (**b**) taken with the polarizing microscope (**C**).

**Figure 2:**
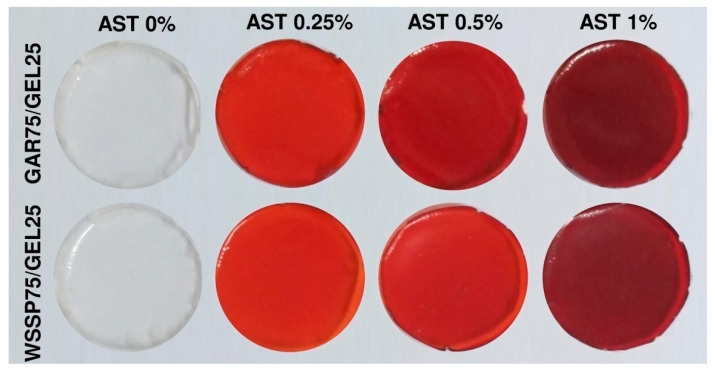
Images of AST-free and AST-supplemented polysaccharide75/GEL25 films.

Microscope images showed good compatibility between the polysaccharides and GEL (lack of aggregates/complexes) (Figure 1A,B), which agreed with our earlier observation [12]. As in previous study [12], the control GAR-based FFS had a uniform and smooth structure, while the WSSP-containing FFS showed a sandy topography (Figure 1B). The globular nature of the WSSP [25] and their limited solubility were probably responsible for the grainy microstructure of the WSSP75/GEL25 FFS. The AST-loaded FFSs and, consequently, the films were intensively red (Figure 2). The AST was more uniformly distributed in the GAR-based system as compared to the WSSP-based FFS (Figure 1B). In the latter carrier, many of the polysaccharide particles were not colorized by AST. It suggests that the WSSP are poorly wettable, which is partially confirmed by the Sw results (Table 1). Since the starch system was a carrier of ASX in the AST formulation, the starch granules, mainly with a diameter of ~ 15–70 µm, were observed in the AST-supplemented FFSs, as evidenced by the presence of Maltese crosses (Figure 1C). The cryogenic treatment of the FFSs resulted in formation of the porous polymeric scaffolds (Figure 1A). As can be seen, there were differences in the pore apertures of the control and AST-supplemented freeze-dried FFSs. It suggests that presence of AST affects process of cryogelation. It is known that incorporation of additional constituents significantly affects the appearance of the polymeric cryogels [6,7]. Lower molecular weight monomers are expected to form larger pores when compared to larger monomers, while an increase in the amount of reaction constituents raises the rigidity of cryogels, thickness of cryogel walls, and elasticity [26]. Interestingly, it was found that the AST could improve the cryo-cross-linking ability of the GAR-based FFS (more interconnected pores were formed) while destroying the structure of the WSSP-based cryo-gel. The observed changes, however, need to be interpreted with caution since other factors, such as temperature and freezing rate (which are not always easy to keep the same), as well as fracture-dependent pore orientation (longitudinal vs. cross-sectional), could significantly affect the quality of the prepared cryo-gels. The observation of the AST-loaded GAR-based cryo-gel showed that the starch grains were anchored in the polymeric scaffolds. Since only a small part of the sample was analyzed using the SEM techniques it was hard to detect and visualize the starch granules in the WSSP75/GEL25 FFS.

### 3.2. FTIR

**Figure 3:**
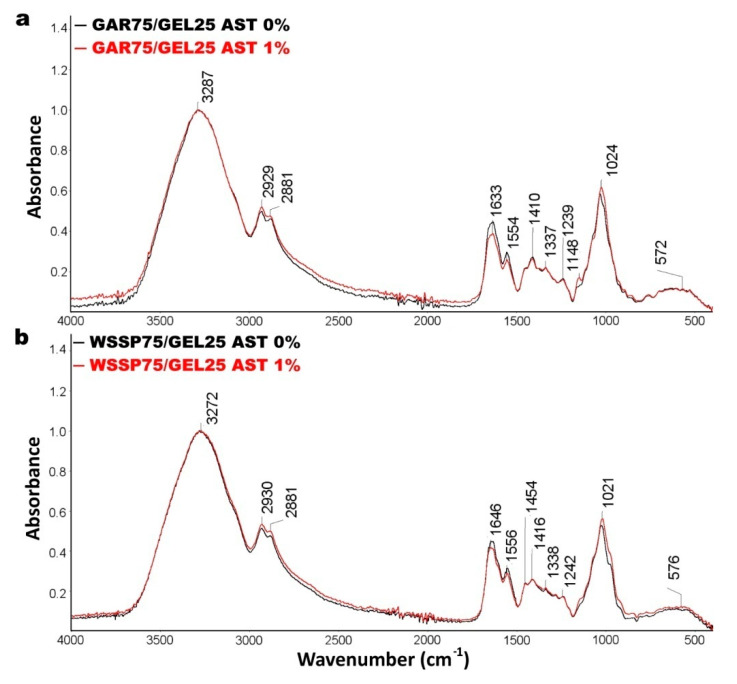
ATR-FTIR spectra of AST-free and 1%AST-supplemented GAR75/GEL25 (**a**) and WSSP75/GEL25 films (**b**).

The infrared spectra of the control and 1%AST-supplemented films are presented in Figure 3. The peaks at 3272 cm^−1^ and 3287 cm^−1^ are attributed to amide A (–OH and/or –NH_2_) stretching. The broad absorption band at ~2930 and ~2880 cm^−1^ represents the stretches in Amide B attributed to the stretching vibrations of asymmetric and symmetric methyl groups (CH_2_). The spectra exhibited an intensive absorption at 1646–1633 cm^−1^ corresponding to the C=O stretching vibration in Amide 1 band. The peak at 1695–1630 cm^−1^ is characteristic of the presence of the –CONH_2_ group, which was most likely formed as the result of the interaction between GEL and polysaccharides [14]. The GAR- and WSSP-based films exhibited the asymmetric stretching vibration of carboxylate at 1554 and 1556 cm^−1^, respectively. The set of vibrations (1454, ~1337, ~1240 cm^−1^, 1148 cm^−1^) is associated with hemicelluloses in the films [27]. The FTIR spectra of all samples showed a strong signal at ~1020 cm^−1^, which suggests the presence of the C–N group [28]. Surprisingly, the spectral profiles of the control and 1%AST-supplemented films were similar, which may be explained by the low amount of AST in the systems. Nevertheless, the AST-supplemented GAR75/GEL25 film showed a small new peak at 1148 cm^−1^ coming from ν (C–O) stretching vibrations. Moreover, it was found that the presence of AST in the GAR- and WSSP-based films slightly decreased the intensity of peaks in the region of 1646–1554 cm^−1^. In accordance with the present results, previous studies have also demonstrated a decrease in the intensity of peaks (e.g., at 1634 and 1548 cm^−1^), which was attributed to the fact that ASX inhibited electrostatic interactions with regard to amino and carbonyl groups within the composite chitosan/GEL film [29].

To date, limited data are available on the possible interactions of the water-soluble ASX with biopolymers. This study was unable to demonstrate the interaction mechanisms between the ASX and the polysaccharide/GEL binary complexes on the molecular level. Nevertheless, based on the fluorescence spectra, the specific interactions between natural ASX and biopolymers (mainly proteins) has been suggested. According to Li and Yan [30], the major part of the action force between ASX and ovalbumin are hydrophobic interactions. In turn, ASX binding to the oleic acid-loaded bovine serum albumin (BSA) complexes is mainly through hydrogen binding and van der Waals interactions [31]. Results of thermodynamic investigations conducted by Li and Li [32] suggested that hydrophobic forces and electrostatic attraction have a significant role in the interaction between ASX and the proteases.

In conclusion, methods other than FTIR should be used in order to get better insight into the possible AST-carrier interactions.

### 3.3. pH, Film Thickness, and Water Affinities

The GAR-based FFSs exhibited higher pH values than the FFSs based on WSSP (Table 1). This result agrees with previous findings [14] and can be explained by the presence of free carboxyl groups of d-glucuronic acid and 4-O-methyl d-glucuronic acid residues in the GAR [33]. As shown by Daoub et al. [34], the pH values for GAR are in the range from 4.45 to 4.94. According to the previous observations [14], the addition of GEL into GAR-based FFS results in reduction of acidity, which explains the pH values (5.15–5.31) observed in this study (Table 1). The incorporation of AST caused acidification of the FFSs. Since the pH of the commercial ASX formulations ranges from 4.50 to 5.00 [35] (i.e., is very similar to the GAR- and WSSP-based FFSs), only a slight decrease in pH was observed.

Since a constant amount of total solids (0.0125 g/cm^2^) was placed on the tray (i.e., with increasing the AST content, the smaller amount of FFSs was cast), the thickness of the films with and without AST was the same (*p* > 0.05, Table 1).

**Figure 4:**
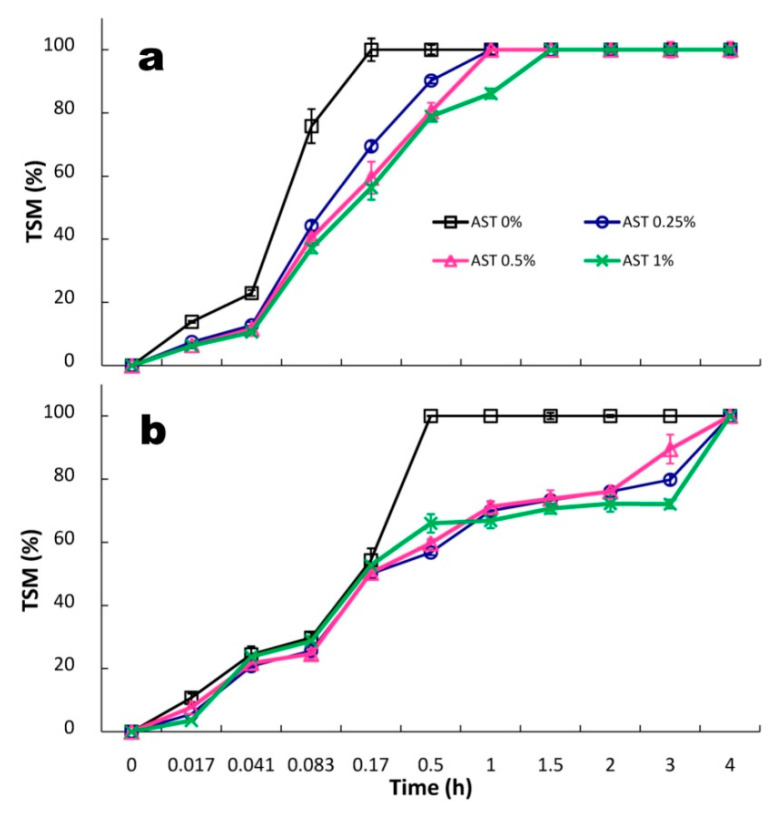
Kinetics of total soluble matter (TSM) of GAR75/GEL25 (**a**) and WSSP75/GEL25 films (**b**) loaded with increasing AST content.

The analysis of water affinities (Table 1, Figure 4) demonstrated that the WSSP75/GEL25 film (less swellable and soluble) was less hydrophilic than the GAR-based carrier. As shown by Madea and Nakamura [36], WSSP contains higher hydrophobic peptide levels than GAR, which may affect its dissolution behavior. Due to the elevated incubation temperature (30 °C), the control GAR- and WSSP-based films were completely soluble after 10 and 30 min, respectively. In turn, the previous study [14] demonstrated that, at a lower dissolution temperature (25 °C), the TSM values of these films were ~82%. The temperature-dependent solubility of GEL (melting point ≈ 32 °C [37]) was likely responsible for the 100% dissolution of the films. The presence of AST in the films reduced the solubility (Figure 4) and the Sw ability (Table 1). There are several possible explanations for this result. It is possible that, due to the presence of starch granules in the films (Figure 1), the water molecules with difficulty penetrated the blend polymeric network. Therefore, as the AST concentration increased, the Sw of the films tended to decrease (Table 1). Additionally, other components of the AST formulation may have developed hydrogen bonds with the bi-polymeric network, which hindered formation of polymer-water hydrogen bonds, resulting in a limited ability to absorb water.

### 3.4. Release of ASX

**Figure 5:**
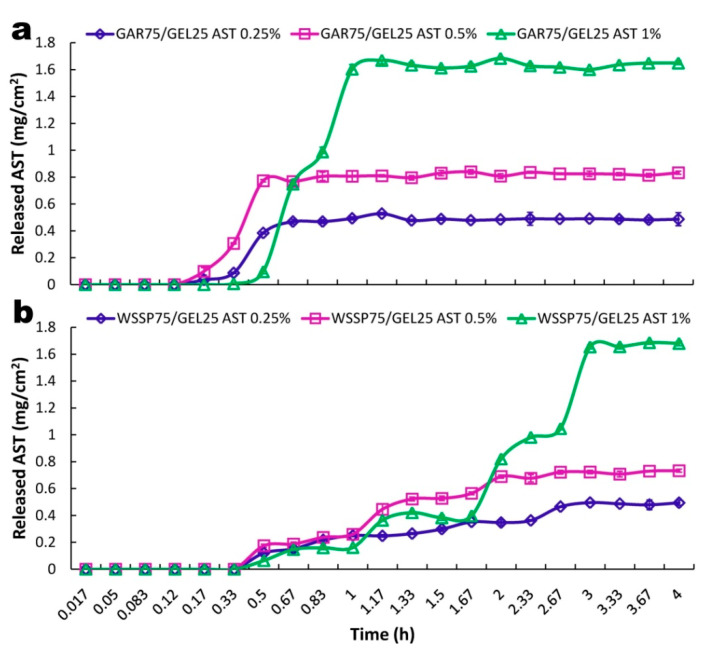
Kinetics of cumulative release (mg/cm^2^) of AST from GAR75/GEL25 (**a**) and WSSP75/GEL25 films (**b**).

**Figure 6:**
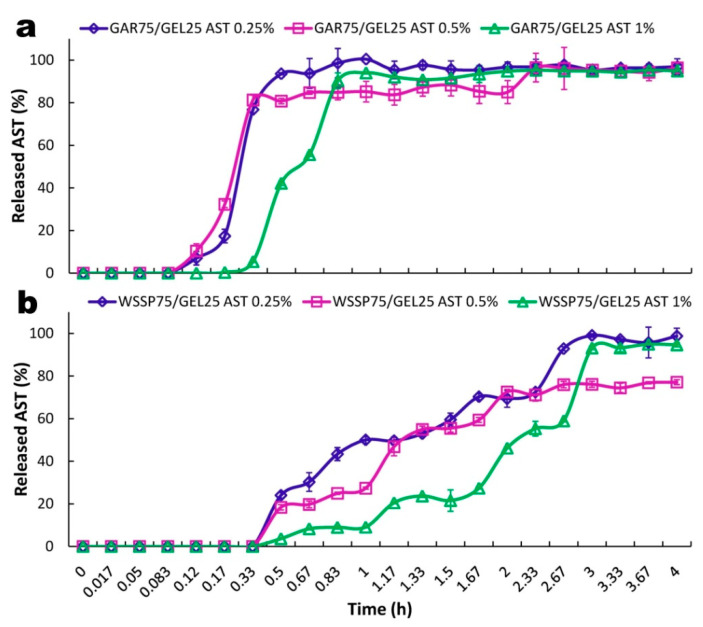
Kinetics of percentage release of AST from GAR75/GEL25 (**a**) and WSSP75/GEL25 films (**b**).

The elevated temperature (30 °C) was used to accelerate AST release from the films. This decision was made based on the preliminary study, which showed that the migration of AST from the carriers at 25 °C was incomplete although the compound was released for several days. The cumulative (mg/cm^2^) amounts of AST released from the GAR- and WSSP-based carriers are shown in Figure 5. In turn, Figure 6 presents the percentage of AST release. For both carriers, the lag times (T_lag_) of the AST release were observed. The WSSP75/GEL25 film ensured a longer T_lag_ (thus, slower release) than the GAR-containing carrier (Figure 5 and Figure 6). The values of t_50%_, calculated according to the best fitting kinetic models, showed that the WSSP75/GEL25 film exhibited ~4 times slower release compared to the GAR-based film (Table 2). Nevertheless, depending on the AST concentration, c.a. 95–97% and 77–99% of the AST was finally released from the GAR- and WSSP-based films, respectively.

Generally, the release of an active substance from a polymer matrix can be categorized in three main processes: (i) diffusion from a non-degraded polymer (diffusion-controlled system); (ii) enhanced drug diffusion due to polymer swelling (swelling-controlled system); (iii) release via polymer degradation and erosion (erosion-controlled system) [38]. Since the carriers used in this study were fully water soluble (at 30 °C), it can be concluded that the AST release was mainly controlled by an erosion-driven process. Therefore, the less erodible character of the WSSP-containing film was probably responsible for the delayed AST release. As can be seen from Table 3, there was a high positive correlation (R^2^ = 0.70–0.96) between the TSM and AST release. 

There is, however, other possible explanation for the delayed release of AST from the WSSP-based carrier. It is known that drug release depends on the branching architecture of the polymers [39]. The atomic force microscopy revealed that WSSP has more highly branched star- or comb-shaped structures in comparison with GAR [40]. In this way, the slower release of AST from the WSSP-based film may be related to stronger physicochemical entrapment of the carotenoid in the matrix with a higher branching degree.

In general, there is a negative correlation between the drug release rate and the matrix swelling rate [41], mainly because a gel network in the swollen state entraps the drug and, thus, increases its diffusion pathway. Nevertheless, in this study, the release of AST from the more swellable matrix system (GAR-based film) was faster than from the less swellable WSSP-based film. It confirms that the matrix swelling was less involved in the AST release. A possible explanation for this might be that at 30°C the swelling of the thin-film carriers was slower than their erosion.

Generally, the increase in the AST concentration resulted in a slower release rate, as indicated by comparison of the t_50%_ values (Table 2). This trend reflects the solubility behavior of the films; i.e., as the AST content increased, the samples tended to be less soluble. 

### 3.5. Mathematical Modeling

**Figure 7:**
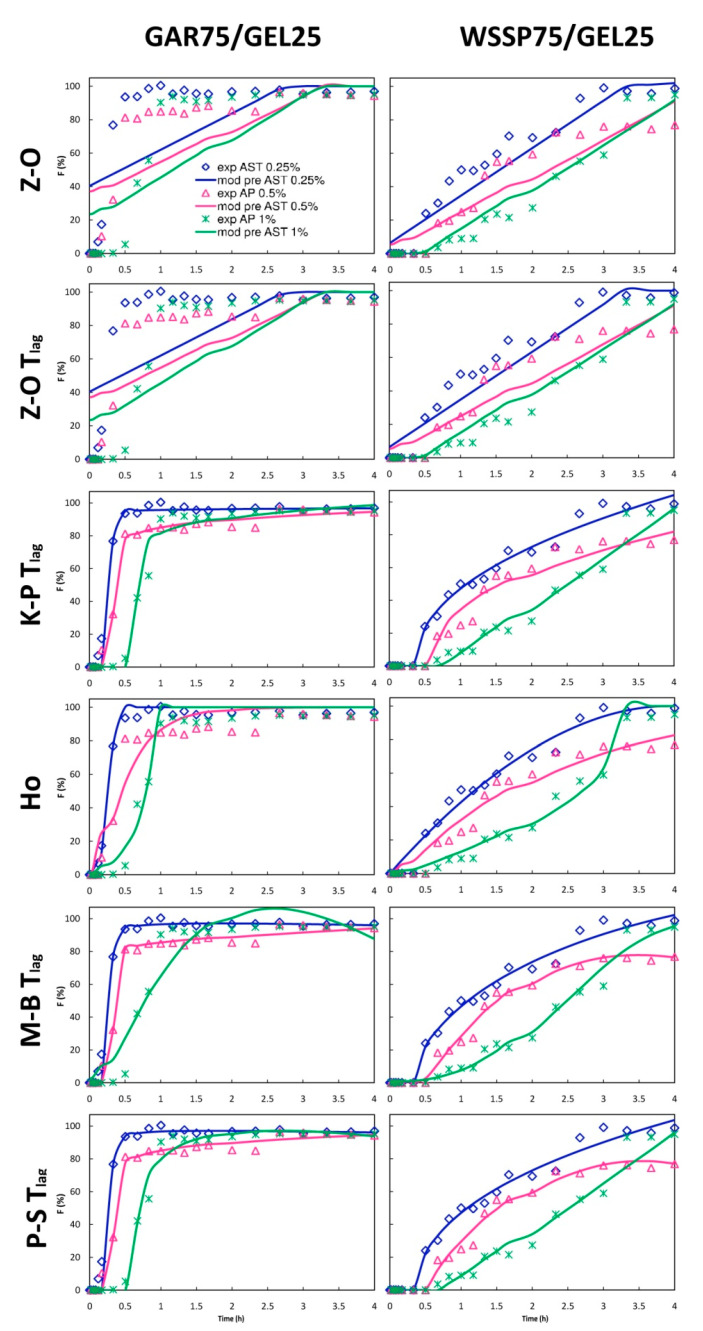
Fraction (F) of AST released from polysaccharide75/GEL25 films according to the Zero-order (Z-O), Zero-order with T_lag_ (Z-O T_lag_), Korsmeyer–Peppas with T_lag_ (K-P T_lag_), Hopfenberg (Ho), Makoid–Banakar with T_lag_ (M-B T_lag_), and Peppas–Sahlin 1 with T_lag_ (P-S T_lag_) models; experimental (exp) and model predicted data (mod pre).

**Figure 8:**
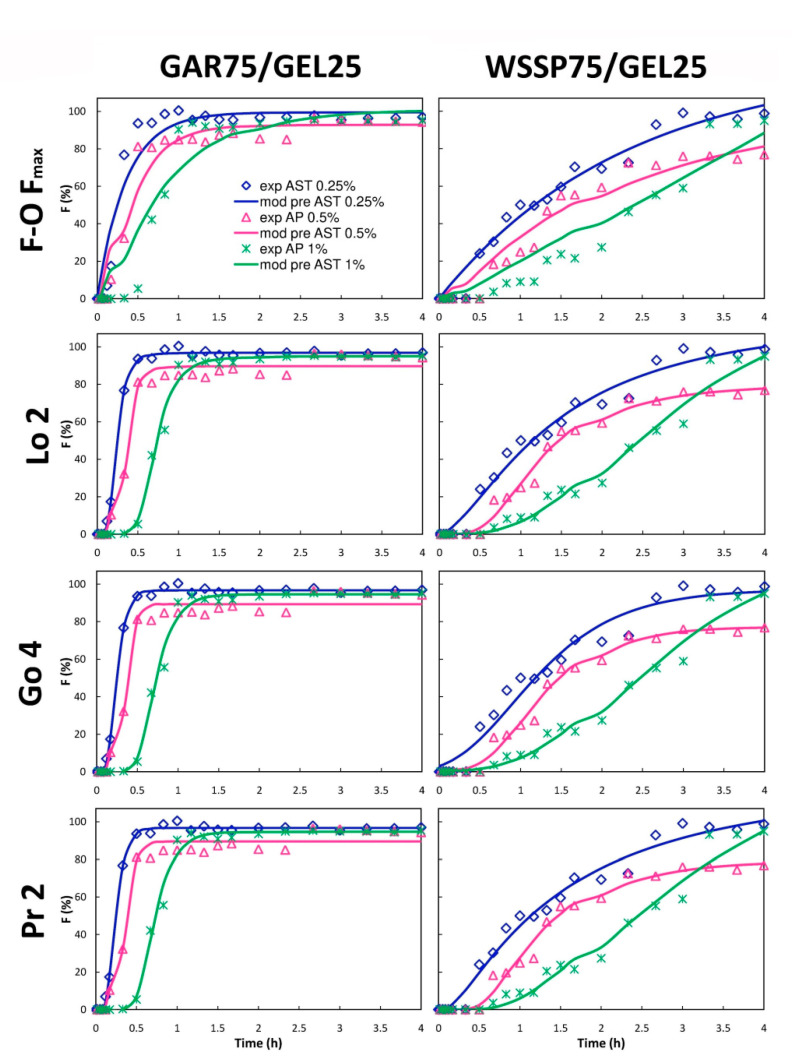
Fraction (F) of AST released from polysaccharide75/GEL25 films according to the First order with F_max_ (F-O F_max_), Logistic 2 (Lo 2), Gompertz 4 (Go 4), and Probit 2 (Pr 2) models; experimental (exp) and model predicted data (mod pre).

In this study, ten mathematical equations (Table S1) were used to determine the kinetics of the AST release from the films (Figure 7 and Figure 8). The identification of the best fitting models with the use of AIC is presented in Table S2, while the parameter values obtained from the mathematical modeling are shown in Table S3 and Table S4. It was impossible to fit one optimal model to describe the migration of AST from the particular carrier types (Table S2). As can be seen from Figure 7 and Figure 8, the WSSP-based carrier had a more linear release profile than the GAR-containing film. Consequently, the Zero-order (Z–O) and Z–O with T_lag_ models had a quite good prediction accuracy for this carrier (Figure 7). The Z–O model describes the system in which a drug is released at a constant rate. Therefore, it can be concluded that the WSSP75/GEL25 carrier may be useful as an extended-release AST system. In particular, the 1% AST-supplemented WSSP75/GEL25 film has the best potential for controlling AST release. According to the Korsmeyer–Peppas with T_lag_ model (K-P with T_lag_), AST in this system was transported via a non-Fickian system (n value between 0.5 and 1), suggesting coupling of the diffusion and erosion release mechanisms (anomalous diffusion). In the non-Fickian type, the swelling or polymeric chain or relaxation is the governing release mechanism, and the migration kinetics obeys the Z–O equation. In the case of the other films, the n values were below 0.5 (n = 0.006–0.451), which was indicative of a quasi-Fickian-controlled mechanism [42]. This suggests that diffusion was the dominant mechanism of AST release from most of the samples. This finding is consistent with that reported by Colín-Chávez et al. [43], who analyzed diffusion of natural ASX from polyethylene active packaging films into ethanol.

### 3.6. Antioxidant Properties

**Figure 9:**
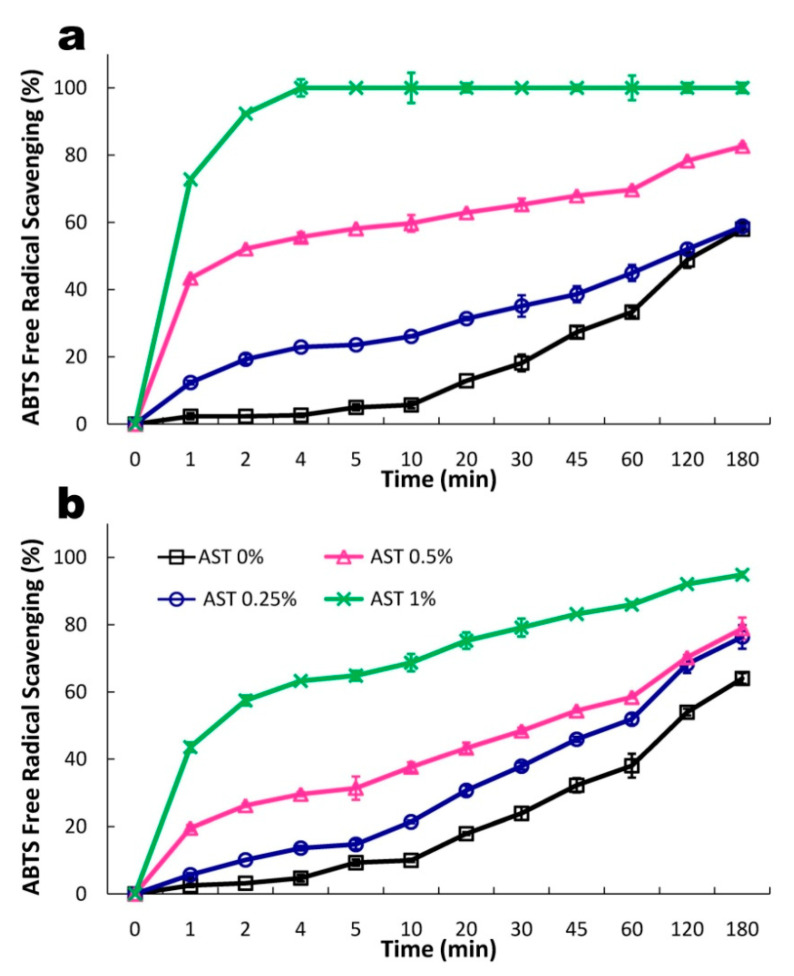
Kinetics of antioxidant activity of GAR75/GEL25 (**a**) and WSSP75/GEL25 films (**b**) loaded with increasing AST content.

Various encapsulating agents have been recently proposed as delivery systems for ASX [44]. It was found that encapsulation of ASX in biopolymers protects it from degradation and improves its bioaccessibility and bioavailability. So far, limited data are available on the effect of biopolymers on the antioxidant activity of ASX. Figure 9 presents the kinetics of the free radical scavenging capacity of the films. Both the bi-polymeric carriers exhibited quite good antiradical activity (Figure 9). The comparison of the T_25%ABTS_ values of the control films revealed that the WSSP75/GEL25 carrier had higher antioxidant potential than the GAR75/GEL25 film (31.23 vs. 42.10 min, Table 2). Some authors link the antioxidant activity of polysaccharides to the bounded protein fraction [45,46]. Following this understanding, it may be assumed that GEL (in spite of its lower concentration in the films) had a greater contribution to the antiradical potential of the carriers than polysaccharides. Numerous studies showed that GEL films exhibited fine antiradical activity [47,48]. The antioxidant properties of proteins are closely related to specific amino acids and/or their sequences, structure, and hydrophobic properties. As reported by Nurilmala et al. [49], fish skin gelatin has higher levels of antioxidants than meat protein due to the high concentration of glycine and proline, which are able to donate electrons, thus, neutralizing free radicals.

A dose-dependent relationship was found between the ABTS^+*^ scavenging activity and the AST content in the films (Figure 9). The addition of AST more efficiently increased the antioxidant potential of the GAR-based film than the WSSP-containing film. Initially (up to 30 min), the GAR-based systems (regardless of the AST concentration), exhibited stronger antioxidant potential than the WSSP-based films, as evidenced by their lower t_25%ABTS_ values (Table 2). This result reflects the AST release profiles (Figure 5), i.e., the faster release rate of the GAR-based film (Figure 6) resulted in stronger antioxidant activity (Figure 9). The data obtained for the longer incubation periods showed that this behavior was noted only for the 0.5 and 1% AST-supplemented films (Figure 9). Surprisingly, the 0.25% AST-supplemented WSSP-based system exhibited higher antioxidant activity than the GAR-containing film. It is difficult to explain this result, but it might be related to the fact that the control WSSP-based carrier had natively higher antioxidant potential compared to the GAR-based film. In the case of most systems (with the exception of the 1% AST-supplemented GAR-based film), a high positive correlation was found between the antioxidant activity and cumulative AST release (R2 = 0.72–0.93; Table 3). Interestingly, weaker correlations between the data were found as the AST concentration increased. It can be assumed that other antioxidants (DL-α-tocopherol and sodium ascorbate) present in the AST formulation altered the antioxidative capacity of the films, especially the systems containing the highest AST load.

## 4. Conclusions

One of the most important characteristics of biopolymers is the possibility to act as carriers to ensure controlled release active of substances. Polymer selection provides the easiest approach by which the active compound migration can be adjusted to the specific needs. The branched polymer systems have the potential to overcome the issues facing immediate-release. As expected, the concept of blending GEL with different branched polysaccharides was found useful in the development of delayed release carrier systems exhibiting a period without AST release (time lag). The WSSP-based carrier (less erodible in water) ensured higher AST trapping efficiency than the GAR-based film (more erodible). Since the WSSP-based carrier demonstrated more proportional AST release rate (close to the Z-O kinetics), it can be assumed that, when this active system is applied in foods (high in moisture content) in the form of coating, casing, or wrap, the AST will persist on the food surface for an extended period of time. The partial immobilization of antioxidant in the packaging material is beneficial since many foods oxidize primarily on the surface.

Likely due to the increasing number of starch granules (from the AST formulation) in the film matrix, the increase in the AST concentration was accompanied by gradually reduced solubility and, consequently, reduced the release rate. This implies that native starch, acting as a potential binder and/or dissolution blocking agent, is helpful in retarding AST release.

A further study should assess the effect of AST on the optical, mechanical, and barrier properties of the obtained films to provide a more useful guide in establishing the best compromise between conflicting demands for film properties.

## Figures and Tables

**Table 1:** Effect of the AstaSana (AST) concentration on pH of film-forming solutions (FFSs), thickness, and swelling (Sw) of 75/25 blend films obtained from polysaccharides: gum arabic (GAR), water-soluble soy polysaccharides (WSSP), and gelatin (GEL).

Film	AST (%)	pH of FFSs	Thickness (µm)	Sw (%)
GAR75/GEL25	0	5.31 ± 0.01 ^g^	85.27 ± 2.77 ^a^	499.40 ± 25.88 ^d^
	0.25	5.28 ± 0.01 ^f^	86.65 ± 3.75 ^a^	416.05 ± 24.58 ^c^
	0.5	5.25 ± 0.01 ^e^	87.65 ± 3.12 ^a^	416.51 ± 20.07 ^c^
	1	5.15 ± 0.00 ^d^	86.96 ± 3.80 ^a^	381.66 ± 15.49 ^c^
WSSP75/GEL25	0	5.01 ± 0.01 ^c^	85.53 ± 3.84 ^a^	301.32 ± 26.52 ^b^
	0.25	4.91 ± 0.01 ^a^	87.09 ± 2.93 ^a^	269.96 ± 18.86 ^ab^
	0.5	4.94 ± 0.00 ^b^	89.90 ± 3.77 ^a^	262.19 ± 28.58 ^a^
	1	4.94 ± 0.01 ^b^	89.02 ± 2.53 ^a^	255.86 ± 14.63 ^a^

^a–g^ Values with the different superscript letters within one column are significantly different (*p* < 0.05).

**Table 2:** Times required for 25% (t_25%_) and 50% (t_50%_) AST release from 75/25 blend films obtained from polysaccharides: gum arabic (GAR), water-soluble soy polysaccharides (WSSP), and gelatin (GEL). The quarter- and half-scavenging times (t_25%ABTS_ and t_50%ABTS_) of the films are shown.

Film	AST (%)	t_25%_ (min)	t_50%_ (min)	R^2^_adjusted_^^^	t_25%ABTS_ * (min)	t_50%ABTS_ * (min)
GAR75/GEL25	0	n.d	n.d	n.d.	42.10	120.76
	0.25	11.28	14.64	0.999 ^(Lo 2)^	7.51	101.47
	0.5	10.2	21.72	0.992 ^(K-P Tlag)^	0.64	1.94
	1	26.46	34.02	0.993 ^(Pr 2)^	0.22	0.53
WSSP75/GEL25	0	n.d	n.d	n.d.	31.23	101.01
	0.25	31.68	65.16	0.988 ^(K-P Tlag)^	12.94	54.95
	0.5	48.48	78.60	0.989 ^(Go 4)^	2.34	30.21
	1	87.54	142.80	0.983 ^(Ho)^	0.91	1.23

^(K-P Tlag)^ obtained from the Korsmeyer–Peppas with T_lag_ model. ^(Ho)^ obtained from the Hopfenberg model. ^(Lo 2)^ obtained from the Logistic 2 model. ^(Go 4)^ obtained from the Gompertz 4 model. ^(Pr 2)^ obtained from the Probit 2 model. ^^^ the goodness of fit of the models. ^*^ data obtained from the Weibull with F_max_ model (R^2^ = 0.990–1.000). n.d.—no data.

**Table 3:** Correlation (R^2^ *) between total soluble matter (TSM) and cumulative AST release (mg/cm^2^) and correlation (R^2^ **) between cumulative AST release (mg/cm^2^) and antiradical activity of the GAR75/GEL25 and WSSP75/GEL25 blend films.

Film	AST (%)	R^2^ *	R^2^ **
GAR75/GEL25	0.25	0.93	0.87
	0.5	0.96	0.83
	1	0.91	0.34
WSSP75/GEL25	0.25	0.89	0.93
	0.5	0.88	0.89
	1	0.70	0.72
All formulations		0.89	0.47

## Data Availability

The data present in this study are available on request from the first author (Katarzyna Łupina).

## Supplementary Materials

The following are available online at https://www.mdpi.com/article/10.3390/polym13071062/s1, Table S1: Mathematical models used to describe the dissolution curves [24], Table S2: Comparison of AIC values obtained from fitting experimental data to the different release models, Table S3: Parameters obtained from fitting experimental data to the release models with the F_max_ parameter, Table S4: Kinetic parameters obtained from modeling without the F_max_ parameter.
