# Supplementary material for: Gum Arabic/Gelatin and Water-Soluble Soy Polysaccharides/Gelatin Blend Films as Carriers of Astaxanthin—A Comparative Study of the Kinetics of Release and Antioxidant Properties
Source: Polymers (Basel). 2021 Mar 28;13(7):1062. doi: 10.3390/polym13071062 (PMC8036643; doi:10.3390/polym13071062)
Supplement: Supplementary file 1 [file polymers-13-01062-s001.pdf]

Table 1. Mathematical models used to describe the dissolution curves [24].

**Model.**	**Equation**	**Parameters**
Zero–order (Z-O)	$F = k_0 \cdot t$	k_0
Zero–order with T_{lag} (Z-O T_{lag})	$F = k_0 \cdot \left(t - T_{lag}\right)$	k_0, T_{lag}
First order with F_{max} (F-O F_{max})	$F = F_{max} \cdot (1 - e^{-k_1 \cdot t})$	k_1, F_{max}
Korsmeyer–Peppas with T_{lag} (K-P T_{lag})	$F = k_{KP} \cdot \left(t - T_{lag}\right)^n$	k_{KP}, n, T_{lag}
Hopfenberg (Ho)	$F = 100 \cdot [1 - (1 - k_{HB} \cdot t)^n]$	k_{HB}, n
Makoid–Banakar with T_{lag} (M-B T_{lag})	$F = k_{MB} \cdot \left(t - T_{lag}\right)\hat{} \cdot Exp\left[-k \cdot \left(t - T_{lag}\right)\right]$	k_{MB}, n, k, T_{lag}
Peppas–Sahlin 1 with T_{lag} (P-S T_{lag})	$F = k_1 \cdot \left(t - T_{lag}\right)^m + k_2 \cdot \left(t - T_{lag}\right)^{2m}$	k_1, k_2, m, T_{lag}
Logistic 2 (Lo 2)	$F = F_{max} \frac{\left[e^{a+\beta \cdot \log(t)}\right]}{1 + e^{a+\beta \cdot \log(t)}}$	α, β, F_{max}
Gompertz 4 (Go 4)	$F = F_{max} \cdot e^{-\beta \cdot e^{-k \cdot t}}$	k, β, F_{max}
Probit 2 (Pr 2)	$F = F_{max} \cdot \Phi[a + \beta \cdot \log(t)]$	α, β, F_{max}
Weibull with F_{max} (Wb F_{max})	$F = F_{max} \cdot \left[1 e^{\frac{(t - Ti)^\beta}{\alpha}}\right]$	α, β, Ti, F_{max}

F is the fraction (%) of drug released in time *t*.

k0 is the Zero-order release constant

T_{lag} is the lag time prior to drug release

k_1 is the First-order release constant

F_{max} is the maximum fraction of the drug released at infinite time

k_{KP} is the release constant incorporating structural and geometric characteristics of the drug-dosage form; *n* is the diffusional exponent indicating the drug-release mechanism

k_{HB} is the combined constant in Hopfenberg model, $k_{HB} = k_0/(C_0 \times a_0)$, where k_0 is the erosion rate constant, C_0 is the initial concentration of drug in the matrix, and a_0 is the initial radius for a sphere or cylinder or the half thickness for a slab; *n* is 1, 2, and 3 for a slab, cylinder, and sphere, respectively

k_{MB}, *n*, and *k* are empirical parameters in Makoid–Banakar model (k_{MB}, n, $k > 0$)

k_1 is the constant related to the Fickian kinetics; k_2 is the constant related to Case-II relaxation kinetics; *m* is the diffusional exponent for a device of any geometric shape which inhibits controlled release

α is the scale factor in Logistic 1 and 2 models; β is the shape factor in Logistic 1 and 2 models

β is the scale factor in Gompertz 4 model; k is the shape factor in Gompertz 4 model

Φ is the standard normal distribution; α is the scale factor in Probit model; β is the shape factor in Probit model

α is the scale parameter which defines the time scale of the process; β is the shape parameter which characterizes the curve as either exponential ($\beta = 1$; case 1), sigmoid, S-shaped, with upward curvature followed by a turning point ($\beta > 1$; case 2), or parabolic, with a higher initial slope and after that consistent with the exponential ($\beta < 1$; case 3)

Ti is the location parameter which represents the lag time before the onset of the dissolution or release process and in most cases will be near zero

Table 2. Comparison of AIC values obtained from fitting experimental data to the different release models.

Film	AST (%)	F-O F_{max}	Lo 2	Go 4	Pr 2	Z-O	Z-O T_{lag}	K-P T_{lag}	Ho	M-B T_{lag}	P-S T_{lag}	Best fit
GAR75/GEL25	0.25	175.26	86.82	87.41	89.11	222.56	222.56	137.44	129.030	139.039	138.932	Lo 2
	0.5	168.83	138.28	139.56	139.18	215.26	215.26	125.69	174.424	127.434	127.607	K-P T_{lag}
	1	180.33	129.37	127.75	127.47	216.02	216.02	151.965	154.789	181.196	143.119	Pr 2
WSSP75/GEL25	0.25	149.35	143.80	154.43	141.47	176.89	176.89	133.377	146.982	133.939	135.041	K-P T_{lag}
	0.5	154.90	126.74	124.75	127.73	178.98	178.98	150.131	155.017	127.992	130.596	Go 4
	1	168.53	142.45	141.21	144.66	160.14	160.14	150.706	137.260	139.295	152.451	Ho

Table 3. Parameters obtained from fitting experimental data to the release models with F_{max} parameter.

Film	AST (%)	F-O F_{max}		Lo 2			Go 4			Pr 2		
		k_1 (1/min)	F_{max}	α	β	F_{max}	k	β	F_{max}	α	β	F_{max}
CMC75/GEL25	0.25	13.286	100.539	4.860	3.776	101.227	21.933	2.569	100.288	2.927	2.258	100.750
	0.5	15.049	97.169	4.129	2.991	98.744	22.563	2.233	96.934	2.535	1.826	97.994
	1	10.310	100.207	4.108	3.471	101.475	17.391	2.511	99.841	2.473	2.072	100.817
OSA75/GEL25	0.25	1.938	64.609	3.486	8.218	62.397	6.621	8.717	62.033	2.051	4.832	62.219
	0.5	2.970	49.637	5.404	7.753	48.157	13.556	10.120	47.818	3.263	4.677	48.025
	1	2.392	36.161	7.706	14.278	34.698	14.686	45.899	34.682	4.449	8.179	34.667

Table 4. Kinetic parameters obtained from modeling without F_{max} parameter. .

Film	AST (%)	Z-O	Z-O T_{lag}		Ho		K-P T_{lag}			M-B T_{lag}				P-S T_{lag}			
		k_0	k_0	T_{lag}	k_{HB}	n	k_{KP}	n	T_{lag}	k_{MB}	n	k	T_{lag}	k_1	k_2	m	T_{lag}
CMC75/GEL25	0.25	37.662	21.656	−1.864	4.379	2.354	97.282	0.051	0.050	114.028	0.173	0.107	0.017	184.585	−82.130	0.264	0.017
	0.5	35.924	21.216	−1.750	0.006	2236.480	93.319	0.082	0.017	106.377	0.148	0.080	0.017	174.082	−75.805	0.238	0.017
	1	35.713	26.421	−0.889	1.092	8.920	95.543	0.079	0.050	115.907	0.225	0.130	0.017	175.899	−74.318	0.307	0.016
OSA75/GEL25	0.25	30.912	28.367	−0.227	0.000	5041.121	59.327	0.067	0.330	69.974	0.215	0.120	0.319	106.128	−44.169	0.310	0.313
	0.5	25.517	23.353	−0.234	0.000	2046.785	45.349	0.077	0.170	58.819	0.396	0.203	0.091	68.554	−21.622	0.103	0.170
	1	24.111	27.090	0.277	0.000	1773.119	34.396	0.026	0.330	35.823	0.060	0.029	0.328	63.175	−28.350	0.149	0.320
